# Long-term neuromuscular consequences of SARS-Cov-2 and their similarities with myalgic encephalomyelitis/chronic fatigue syndrome: results of the retrospective CoLGEM study

**DOI:** 10.1186/s12967-022-03638-7

**Published:** 2022-09-24

**Authors:** Frédérique Retornaz, Stanislas Rebaudet, Chloé Stavris, Yves Jammes

**Affiliations:** Department of Internal Medicine and Infectious Diseases, European Hospital in Marseille, 6 rue Desirée Clary, 13003 Marseille, France

**Keywords:** Long-COVID, Myalgic encephalomyelitis/chronic fatigue syndrome, Evoked myopotentials, Neuromuscular symptoms, Exercise performance, Handgrip strength

## Abstract

**Background:**

Patients with long-COVID often complain of continuous fatigue, myalgia, sleep problems, cognitive dysfunction, and post-exertional malaise. No data are available on EMG recording of evoked myopotentials (M-waves) or exercise-induced alterations in long-COVID patients, providing evidence of muscle membrane fatigue. Myalgic encephalomyelitis/chronic fatigue syndrome (ME/CFS) develops in more than half of patients after an infectious disease, particularly viral diseases. A large proportion (around 70%) of these patients have neuromuscular disorders with M-wave alterations during and after exercise. Our hypothesis was that M-wave alterations would be also found in long-COVID patients, in association with neuromuscular symptoms, similar to ME/CFS.

**Methods:**

This retrospective observational ColGEM (Covid LonG Encéphalomyelite Myalgique) study compared 59 patients with long-COVID and 55 ME/CFS patients with a history of severe infection who presented before the COVID pandemic. All of these patients underwent the same protocol consisting of a questionnaire focusing on neural and neuromuscular disorders and M-wave recording in the rectus femoris muscle before, during, and 10 min after a progressive cycling exercise. Maximal handgrip strength (MHGS) and maximal exercise power were also measured. The frequency of symptoms and magnitude of M-wave changes in the two groups were compared using non-parametric and parametric tests.

**Results:**

The frequency of fatigue, myalgia, sleep problems, cognitive dysfunction, and post-exertional malaise as well as the magnitude of exercise-induced M-wave alterations were the same in the two groups. By contrast, digestive problems were less present in long-COVID. M-wave alterations were greater in ME/CFS patients as in those with long-COVID when the highest muscle strength and highest exercise performance were measured.

**Conclusions:**

These high clinical and biological similarities between long-COVID and ME/CFS support the hypothesis that SARS-Cov-2 infection can cause ME/CFS symptoms.

*Trial registration* Registered retrospectively.

## Background

SARS-Cov-2 infection is well documented and often includes fatigue and myalgia [1–4]. A meta-analysis by Bornstein et al. [5] showed that a large number of individuals infected with SARS-CoV-2 complain of continuing fatigue many months after the onset of the disease. This phenomenon has been termed “post-COVID syndrome” or “long-COVID” and defines a series of chronic symptoms that patients experience after resolution of acute SARS-Cov-2 infection.

Few clinical studies have reported the neuromuscular consequences of SARS-Cov-2 after 6 months. A retrospective Chinese study of 1733 patients by Huang et al. [6] focused on the evolution of symptoms and health-related quality of life. At 6 months after the initial acute infection, fatigue and muscle weakness were present in 63% of patients and the 6-min walking distance was below the normal range in 75%. Baig et al. [7] reported the adverse effects of SARS-Cov-2 infection on the central nervous system in patients with chronic SARS-CoV-2 infection, however no measurements of muscle membrane excitability have been performed in patients with long-COVID.

A meta-analysis of clinical studies of patients with long-COVID suggests many overlaps with the clinical presentation of another chronic disease, namely the myalgic encephalomyelitis/chronic fatigue syndrome (ME/CFS) [8].ME/CFS is an illness characterized by persistent fatigue at rest which is made worse by exercise [9]. ME/CFS often follows severe bacterial or viral infection by various pathogens, including Epstein-Barr virus, cytomegalovirus, human herpes virus, enterovirus, parvovirus, and mycoplasma [10–14]. Our hypothesis was that ME/CFS may also occur after SARS-Cov-2 infection.

It has already been shown that a large proportion (around 70%) of patients with ME/CFS have neuromuscular disorders characterized by exercise-induced alterations of muscle excitability, assessed by decreased amplitude and lengthening of M-waves [14–17]. The aim of this study was to investigate whether similar exercise-induced M-wave alterations occur in patients with long-COVID. The data were compared to a group of ME/CFS patients with a history of severe infection, selected prior to the COVID pandemic.

## Methods

### Study population

The retrospective CoLGEM (Covid LonG Encéphalomyelite Myalgique) study compared 59 patients suffering from chronic SARS-Cov-2 infection (long-COVID) for at least 6 months and 55 ME/CFS patients with a prior history of severe viral or bacterial disease. ME/CFS patients were all selected from the period before the COVID pandemic; those who had suffered from fatigue for more than 3 years were excluded.

### M-wave recording

All patients underwent the same protocol consisting of the recording of M-waves in the rectus femoris muscle at rest, during an incremental cycling exercise reaching 80% of maximal predicted power, and after a 10 min post-exercise recovery period. This protocol has been performed routinely in our department for more than 6 years in order to identify and characterize neuromuscular alterations in patients with chronic fatigue.

### Exercise protocol

The protocol used to investigate the exercise potential in patients with ME/CFS has been published previously [14–16]. The exercise trial was performed on an electrically braked cycle ergometer (eBike General Electric, USA) driven by microcomputer software (GE Healthcare, Merignac, France). The load was increased at a rate of 20 W/min until fatigue forced the subject to stop the exercise session; the subject then continued to pedal for the first 2 min of a 10 min recovery period. Percutaneous O_2_ saturation was measured continuously (Nellcor model N3000; Kansas City, TX, USA). Twelve ECG leads were recorded and heart rate was monitored continuously. Arterial pressure was measured using a sphygmomanometer.

### Maximal handgrip strength (MHGS)

MHGS was measured in the seated position, with the wrist in the neutral position to hold the handgrip device (model 5401; Takei Scientific Instruments Co. Ltd., Niigata-City, Japan). Study participants were instructed to perform three maximal handgrips sustained for 3 s. The highest MHGS of the three contractions, expressed in Newtons (N), was considered the maximum. Each forearm was tested. The reference values were those reported by Steiber [18].

### EMG recording and analysis

Bipolar (30 mm centre-to-centre) Ag–AgCl surface electrodes (Medtronic, 13 L 20 Skovlunde, Denmark) were used to measure EMG voltage from the rectus femoris muscle on the dominant side of the body. The electrodes were placed between the motor point and the proximal tendon. Inter-electrode impedance was kept below 2000 X by careful skin shaving and abrasion with an ether pad. The EMG signal was amplified (Nihon Kohden, Tokyo, Japan; common mode rejection ratio, 90 dB; input impedance, 100 mX; gain, 1000–5000) with a frequency band ranging from 10‒2000 Hz. Compound muscle mass action potentials (M-waves) were evoked by direct muscle stimulation, using a monopolar technique [14–16]. A constant-current neurostimulator (Grass, Quincy, MA, USA) delivered supramaximal shocks with 0.1-ms rectangular pulses through an isolation unit. One small (1 × 1 cm) negative silver electrode was applied on the main motor point of the muscle and a large (3 × 3 cm) positive silver electrode was placed on the opposite side of the thigh. Supramaximal stimulation was defined as the pulse intensity level approximately 15% above the level yielding an M-wave of maximal amplitude. The signal was fed to an oscilloscope (model DSO 400; Gould, Ballainvilliers, France) and the mean value of the M-waves from eight successive potentials was calculated and use to determine the peak M-wave amplitude and duration.

### Statistical analyses

Intergroup differences between the frequencies of clinical symptoms were determined using the Mann–Whitney rank sum test and ANOVA was used to determine intergroup differences between cardiovascular, handgrip strength, and M-wave values. Linear regression was used to investigate the changes in M-wave amplitude and duration, symptom duration, MHGS, and maximal exercise power. With the numbers available, significant differences were indicated by a p < 0.05.

## Results

### Study population

Mean age and sex ratio were the same in the 2 groups (ME/SFC vs. long-COVID: 46 ± 3 vs. 46 ± 2 years, 77% vs. 73% female, respectively) (Table 1). Fatigue onset began significantly earlier in ME/CFS patients compared to long-COVID patients, who were investigated as close as possible to the acute SARS-Cov-2 infection. As shown in Table 1, the incidence of symptoms was similar in the two groups. Except for digestive problems, which were significantly (p < 0.05) less frequent in long-COVID patients, the criteria for ME/CFS were found in the majority of patients infected with SARS-Cov-2 (69–81%).

**Table 1:** Incidence of symptoms in patients

	ME CFS patients	Long term SARS-Cov-2
	N = 55	N = 59
Age (year)	46 ± 3	46 ± 2
Weight (kg)	66 ± 2	72 ± 2
Women	43 (77%)	43 (73%)
Fatigue onset (months)^a^	34 ± 3	18 ± 3
Myalgia	50/55 (91%)	45/59 (76%)
Sleep problems	45/55 (82%)	48/59 (81%)
Cognitive dysfunction	48/55 (87%)	48/59 (81%)
Digestive problems^a^	47/55 (85%)	37/59 (63%)
Post exertional malaise	48/55 (87%)	43/59 (78%)
Psychological stress	41/55 (75%)	41/59 (69%)

^a^Significant difference (p < 0.05)

Table 2 shows that exercise performance was the same in the 2 groups of patients; pedaling was always interrupted by fatigue. MHGS was similar in the 2 groups but was slightly lower than the lower limit of normal value (i.e. 300 N).

**Table 2:** Response to exercise and neuromuscular alterations in Long-Covid and ME CFS patients

	ME CFS	Long-Covid
Max work rate (W)	117 ± 4	110 ± 4
Max work rate (%)	87 ± 3	80 ± 2
HR max (bps)	142 ± 2	145 ± 6
Systolic BP max (mmHg)	163 ± 8	167 ± 4
Diastolic BP max (mmHg)	86 ± 2	90 ± 4
Handgrip max (N)	284 ± 1 (95%)	278 ± 10 (91%)
Mwave change:	35/55 (64%)	24/59 (41%)
Delta Mw amplitude	− 47 ± 4%	− 41 ± 4%
Delta Mw duration	+ 22 ± 4%	+ 15 ± 3%

**Fig. 1:**
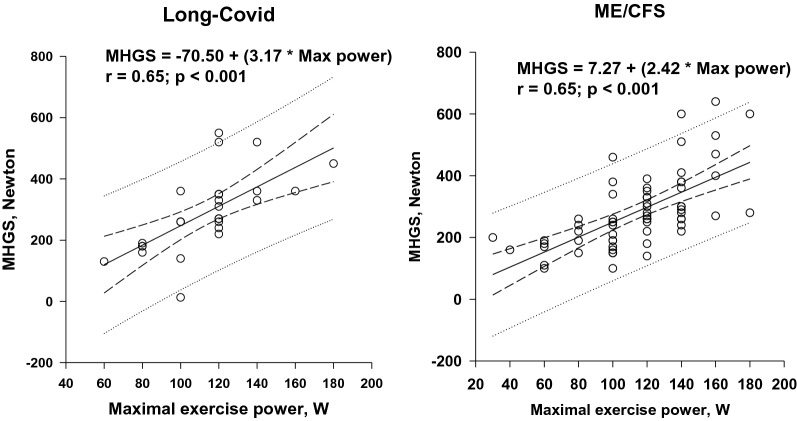
Linear relationships [95% CI] between maximal handgrip strength (MHGS) and maximal cycling exercise power. Regression equations are shown

**Fig. 2:**
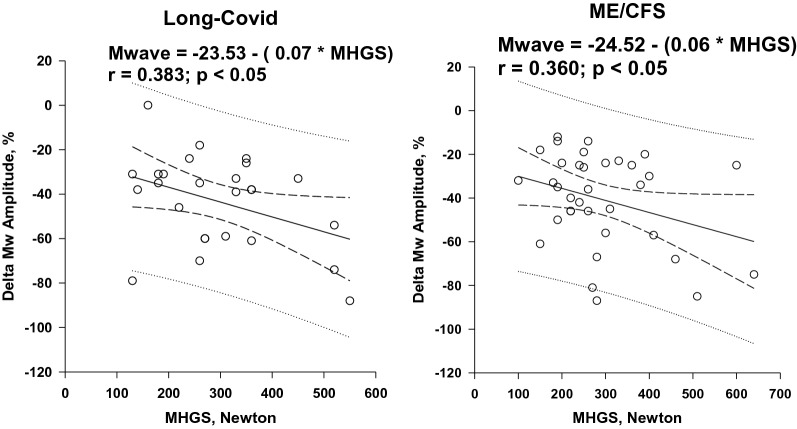
Linear relationships [95% CI] between exercise-induced changes in M-wave amplitude and maximal handgrip strength (MHGS). Changes in M-wave amplitude are expressed as a percentage of resting values. Regression equations are shown

Table 2 shows that M-wave changes were present in both groups. The decrease in M-wave amplitude and duration of lengthening was the same (M-wave amplitude decrease of 47% vs. 41% in ME/CFS vs. long-COVID, respectively). There was no link between long-COVID duration and magnitude of M-wave alterations. In both groups, MHGS was positively correlated with maximal exercise power (Fig. 1) and negatively correlated with the magnitude of M-wave changes (Fig. 2). Thus, M-wave alterations were greater in long-COVID patients with higher muscle strength and higher exercise performance.

## Discussion

The results of this study reveal impressive similarities between the incidence of neural and neuromuscular symptoms in patients with long-COVID and ME/CFS. The only difference was a significantly lower incidence of digestive problems in long-COVID patients than in ME/CFS patients, but the frequency of myalgia, sleep problems, cognitive/memory dysfunction, and post-exertional malaise was the same. The study also shows that despite fatigue onset beginning much earlier in ME/CFS patients, cycling exercise performance and MHGS, a valid index of maximal physical performance [19], were also similar. No intergroup differences in the frequency of M-wave alterations during and after exercise or the magnitude of the changes were observed. In both groups, the magnitude of M-wave alterations was greater in patients with higher exercise performance. The relationship between MHGS and maximal exercise power was confirmed, as reported previously in other ME/CFS patients and healthy individuals [19].

Clinical similarities between long-COVID and ME/CFS have already been reported. Perrin et al. [20] proposed that a proportion of patients infected with SARS-Cov-2 may go on to develop severe “post-COVID-19 syndrome” characterized by long-term adverse events resembling ME/CFS symptoms, such as persistent fatigue, diffuse myalgia, depressive symptoms, and non-restorative sleep. A systematic review by Wong et al. [8] analyzed 29 studies comparing long-COVID symptoms with a list of ME/CFS symptoms. Twenty-five of these 29 studies reported ME/CFS symptoms, suggesting many overlaps with the clinical presentation of ME/CFS. In their clinical analysis, Komaroff et al. [21, 22] also reported that insights from ME/CFS may help to unravel the pathogenesis of post-acute COVID-19 syndrome. Paul et al. [23] proposed many steps that clinicians can take to improve the health, function, and quality of life of patients with ME/CFS, including those in whom ME/CFS develops after SARS-Cov-2. In a 2021 audio interview, Antony Fauci said that “patients post-COVID-19 may develop a post-viral syndrome that is strikely similar to myalgic encephalomyelitis/chronic fatigue syndrome” [24].

We understand that the present study fails to demonstrate some analogies between blood biomarkers measured in long-COVID and ME/CFS patients. Wood et al. [25] reviewed the current literature on the role that mitochondria, oxidative stress, and antioxidants play in our understanding of the pathophysiology of chronic fatigue and SARS-Cov-2.This meta-analysis revealed that increased oxidative stress and systemic inflammation can both be detected in ME/CFS patients [14–16] and in those suffering from long-COVID [25]. However, few studies have reported that the other biological disorders are present in ME/CFS and long-COVID patients. Endothelial biomarkers are inconstantly altered in ME/CFS and long-COVID patients. Hoffke et al. [26] found that only 5 of their 14 post-COVID ME/CFS patients and five of their 16 ME/CFS patients showed endothelial dysfunction defined by a diminished reactive hyperaemia index using peripheral arterial tonometry. Moreover, in the present study, the cardiovascular response to exercise (maximal increases in systolic and diastolic blood pressure) did not differ between ME/CFS and long-COVID groups (Table 2). We already showed that the cardiovascular response to exercise did not significantly differ between healthy individuals and ME/CFS patients [15].

The major strength of this study is the selection of ME/CFS patients based on a report of serious infection preceding fatigue onset. In the majority of these patients (73%), a history of glandular fever with a high level of anti-Epstein-Barr antibodies was noted. All of these ME/CFS patients were investigated in the period preceding the SARS-Cov-2 pandemic (between February and December 2019).

## Conclusions

A number of similarities exist between long-COVID and ME/CFS. Since ME/CFS often follows a viral illness it is tempting to speculate that SARS-Cov-2 infection may cause ME/CFS. Future studies are warranted to confirm these findings.

## Data Availability

The datasets used and/or analyzed during the current study are available from the corresponding author upon reasonable request.

## Abbreviations

ANOVA: Analysis of variance
ColGEM: Covid LonG Encéphalomyélite Myalgique
ME/CFS: Myalgic encephalomyelitis/chronic fatigue syndrome
MHGS: Maximal handgrip strength
M-wave: Evoked myopotential
